# Predictive value of Cmmi-MHR combined with thromboelastography parameters in acute cerebral infarction

**DOI:** 10.1186/s12880-024-01299-0

**Published:** 2024-05-18

**Authors:** Zhongxian Rao, Wei Tan, Junmin Wang, You Zhou, Xue Yang, Shanshan Hu

**Affiliations:** 1 Geriatric Hospital Affiliated to Wuhan University of Science and Technology, Wuhan, Hubei 430065 China; 2 General Medicine, Geriatric Hospital Affiliated to Wuhan University of Science and Technology, Wuhan, Hubei 430065 China; 3 Neurosurgery, People’s Hospital of Wuhan University, Wuhan, Hubei 430060 China; 4 Neurosurgery, Tianyou Hospital Affiliated to Wuhan University of Science and Technology, Wuhan, Hubei 430064 China; 5 General Medicine, Emergency Department, Wuhan University of Science and Technology Hospital, Wuhan, Hubei 430065 China

**Keywords:** Acute cerebral infarction, High frame rate imaging, Predictive value, Monocyte to high-density lipoprotein ratio

## Abstract

Cerebral infarction is a common neurological disease with high rates of morbidity, mortality, and recurrence, posing a great threat to human life and health. Cerebral infarction is the second leading cause of death in the world and the leading cause of long-term disability in humans. The results of the third national retrospective sampling survey on causes of death in 2008 showed that cerebral infarction has become the leading cause of death in China and its mortality rate is 4–5 times that of European and American countries. Therefore, this article proposed a study on the predictive value of Cmmi-MHR combined with thromboelastography parameters that was performed for acute cerebral infarction. This paper mainly proposed a high frame rate imaging technology and analyzed its algorithm. In this article, in the experimental part, an in-depth analysis of the predictive value of the Monocyte-to-high-density lipoprotein cholesterol ratio (MHR) combined with thromboelastography parameters was performed for acute cerebral infarction. The final experimental results showed that HDL (OR = 1.695%, *P*-trend = 0.049) had a probability of death within 90 days of hospitalization (OR = 0.81, 95% CI = 1.06–3.11, *P*-trend = 0.523). There were no significant differences in mortality rate after 90 days. Regardless of adjusting for confounders such as age, gender, and NIHSS score, there was no significant difference in the risk of MHR or monocyte count within 90 days of hospitalization. The conclusion indicates that the combination of Cmmi-MHR and thromboelastography parameters provides a new perspective and method for the diagnosis and treatment of cerebral infarction, and provides important support for personalized treatment and management of cerebral infarction.

## Introduction

A large survey was conducted in 13 provinces around the country, and the results showed that cerebral infarction was the third leading cause of death among men, while cerebral infarction accounted for 21.6% of men and 20.8% of women. Currently, there are 1.5 million patients in China who die of cerebral infarction every year. 80% to 85% of patients have ischemic cerebral infarction, and large artery atherosclerosis and cardiogenic factors are the main factors causing ischemic cerebral infarction. The new inflammatory index MHR is a new inflammatory index, and the increase in MHR has a great relationship with the occurrence of cardiovascular events. Cmmi-MHR is an indicator used to evaluate cerebral collateral circulation and hemodynamic status in patients with cerebral infarction. It can help doctors evaluate the stability of collateral circulation and hemodynamic risks of patients, providing an important basis for treatment decisions. It is calculated by combining the stability of the glial images with the average hemodynamic risk parameters. The stability of the glial images reflects the protective effect of collateral circulation, while the average hemodynamic risk evaluates the patient’s blood flow status. A lower Cmmi-MHR value indicates stable collateral circulation and lower hemodynamic risk, making it suitable for treatment methods such as thrombolysis to reconstruct blood flow. A higher value of Cmmi-MHR can indicate unstable collateral circulation and a higher hemodynamic risk for patients, requiring the consideration of more conservative or invasive treatment strategies. Prediction of acute cerebral infarction through the combination of Cmmi-MHR and thromboelastography parameters has important practical value in improving the effectiveness and quality of cerebral infarction treatment and improving the prognosis of patients.

The article focuses on the high frame rate imaging technology that gives the corresponding algorithm, and then deeply studies the application of MHR and thromboelastography parameters in acute cerebral infarction. The innovation of this paper was that it not only analyzed the high frame rate imaging technology but also extended algorithms, respectively, which can better pave the way for the technology in the experimental part.

Finally, the main contributions of work are as follows:

1. Determine diagnostic indicators: This article has identified Combining Cmmi-MHR and thromboelastography parameters as indicators to evaluate the prognosis of acute cerebral infarction. Combining Cmmi-MHR and thromboelastography parameters provides a reliable measurement standard for the diagnosis of acute cerebral infarction.
2. Improving diagnostic accuracy: The combination of Cmmi-MHR and thromboelastography parameters can significantly improve the diagnostic accuracy of acute cerebral infarction. Compared to individual clinical indicators or traditional imaging evaluations, this combined parameter can more accurately identify patients with cerebral infarction and predict their likelihood of disease progression.
3. Provide new diagnostic tools: This article introduces the Cmmi-MHR combined with the thrombus elastic high frame rate imaging algorithm, which provides a more comprehensive assessment of the risk of cerebral infarction. It can help doctors identify acute cerebral infarction patients early, take more effective treatment measures, and improve the effect of treatment and the survival rate of patients.
4. Enriching research areas: The results of the retrospective analysis of this article enrich the research field of prognosis evaluation for acute cerebral infarction, providing new directions for further exploration of the diagnosis and treatment of cerebrovascular diseases. The application of Cmmi-MHR combined with thromboelastography parameters provides valuable experience and reference for future related research.

In the organizational structure of this article, Chapter 2 mainly introduces the research status of Cmmi-MHR combined with thromboelastography parameters and acute cerebral infarction imaging; Chapter 3 mainly introduces the implementation mechanism of imaging algorithms; Chapter 4 mainly introduces the experimental design, results, and discussion of retrospective research; Chapter 5 summarizes the conclusion of this article.

## Related work

Overexpression of inflammasomes and inflammatory cytokines is the main factor that leads to the occurrence and repair of acute cerebral infarction. The objective of Zhu H was to evaluate the activity of paraoxonase 1 (PON1) and markers of oxidative / antioxidant stress in acute cerebral infarction [1]. LUO Bin studied and analyzed the relationship between the serum high mobility group box protein B1(HMGB1) and the glial fibrillary acid protein (GFAP) and the severity of acute cerebral infarction(ACI) and their predictive value for prognosis [2]. Zhao M explored the predictive value of the neutrophil-to-lymphocyte ratio (NLR) in the prognosis of patients with acute cerebral infarction [3]. Wang Q aimed to investigate the expression and significance of missing melanoma 2 (AIM2) in the plasma of patients with acute cerebral infarction [4]. However, they did not conduct an in-depth analysis of their imaging technology and did not provide technical references. The basic principle of the predictive value of combining Cmmi-MHR with thromboelastography parameters is to comprehensively assess the risk and severity of cerebral infarction by combining the stability of collateral circulation with the elastic characteristics of thrombotic tissue. Yuan Q evaluated the thromboelastography values of patients with acute cerebral infarction and their correlation with routine examinations. The TEG5000 thromboelastography system was used to obtain the TEG parameters, and the research results showed that the TEG parameters are sensitive indicators of hypercoagulability in patients with acute cerebral infarction [5]. Shi Z studied the application of thromboelastography in predicting early neurological deterioration in patients with acute ischemic stroke upon admission and its potential correlation with the evolution of ischemic lesions. The results showed that the shorter time to TEGR at admission in patients with acute ischemic stroke was associated with neurological deterioration in 3 days [6]. Roh D confirmed functional coagulation differences by thromboelastography and evaluated the correlation between the location of cerebral hemorrhage (independent variable) and the results of the TEG and traditional plasma coagulation test (dependent variable) using a linear regression model. The results showed that compared to lobar cerebral hemorrhage, the deep TEG R time was longer (0.57 min; 95% confidence interval: 0.02–1.11; *P* = 0.04), indicating a longer time for thrombosis formation [7]. Current research using thromboelastography provides effective support for the diagnosis and treatment of brain diseases, but thromboelastography is usually limited to specific vascular sites or lesions, and observation of some deep brain structures or blood vessels may be difficult.

Optical sensing using single-photon-counting avalanche diode detectors has become a versatile method for ranging and low-light imaging. Godinho T M proposed a method to create a simulated medical imaging repository based on the indexing of model datasets, the extraction of patterns, and the modeling of research production [8]. Jaros M introduced the acceleration of the k-means algorithm for image segmentation. This speed-up was achieved through efficient parallelization [9]. Qasim A F conducted an exhaustive survey on medical image watermarking, clarifying the requirements for medical image watermarking and the use of medical images [10]. Although all described acute cerebral infarction and its predictive value, they did not integrate imaging techniques with it.

## High frame rate imaging algorithm

### High-frame rate imaging

In a conventional ultrasound system, B ultrasound images are all linear scans and the number of transmissions is the same as the number of beams that make up a scan line. Therefore, the frame rate of conventional ultrasound images is limited by the ultrasound transmission rate, which can only reach 30–40 frames per second [11, 12]. Improving the frame rate of images is of great significance for the development of medical ultrasound diagnostic technology. In this paper, real-time 3D ultrasound technology may be used to more accurately track the movement of the heart, evaluate its function, and use its fine visualization effect on transient processes to indirectly reflect nonstructural information such as mechanical effects and electrical action potentials [13].

### The impact of imaging on acute cerebral infarction

Acute cerebral infarction imaging is the use of high-frame-rate medical imaging techniques to detect and diagnose if a patient has acute cerebral infarction, as well as to evaluate the degree of brain tissue damage and the location of the infarct. It can help doctors in timely diagnosis and treatment, thus minimizing the damage of cerebral infarction to patients as much as possible. The effect of imaging directly affects the doctor’s diagnosis of diseases and the choice of treatment plans, especially for acute diseases such as acute cerebral infarction. Therefore, high-quality imaging technology and accurate imaging results are crucial to improving the accuracy of medical diagnosis and treatment effectiveness. The main factors affecting the imaging effect of acute cerebral infarction include:

1. Timing

Scheduling has a direct and important impact on the effectiveness of imaging diagnosis. Especially in cases of acute ischemic stroke. In this case, the timing of the imaging is crucial as the window for detecting imaging anomalies may be limited. If the imaging is performed too early, such as shortly after the onset of symptoms, it can lead to incomplete formation of the infarct, making the abnormal changes not significant enough, thereby affecting the precision of diagnosis. On the other hand, if imaging is delayed, even if the infarct has already formed, irreversible brain tissue damage may occur, leading to reduced treatment effectiveness and even increasing the patient’s risk of disease deterioration and death.

(2) Size and location of the inflection

The size of the infarction directly affects the sensitivity and resolution of the imaging, and smaller infarcts may not be easily detected in imaging, especially in more conventional imaging techniques such as head CT. In addition, larger infarcts are usually easy to observe and identify, but may also produce artifacts or partially occlude surrounding structures in imaging, affecting the accuracy of diagnosis. The location of the infarction also has a significant impact on the diagnostic imaging effect. Different regions of the brain may be affected by the sensitivity and resolution of imaging techniques, making it difficult to detect or recognize infarcts in certain areas.

(3) Imaging Modality

Different imaging methods have different principles and characteristics and are suitable for different types of lesions and clinical situations. For example, head CT is a conventional imaging method suitable for rapid diagnosis of emergencies such as acute cerebral infarction, but its resolution is relatively low and may not accurately display small lesions. On the contrary, brain magnetic resonance imaging has a higher resolution and excellent display effect in soft tissues, making it suitable for more detailed evaluation of brain anatomy and lesions, especially for the early diagnosis of cerebral infarction. In addition, cerebral angiography technology can provide a visual display of cerebral vascular structure and blood flow, helping to assess the condition of vascular stenosis or obstruction. Cerebral perfusion imaging can evaluate cerebral blood flow and perfusion status, providing important information to assess the severity and prognosis of cerebral infarction.

(4) Image segmentation

Image segmentation is the process of dividing an image into different regions or objects, which can help physicians accurately locate and identify the areas of the lesion, improving the accuracy and reliability of diagnosis [14]. In medical imaging, image segmentation can be used to separate brain tissue, blood vessels, and lesion areas in brain images, helping physicians to observe and analyze the anatomical structure of the brain and the lesion features of the patient more clearly [15, 16]. Through image segmentation, physicians can more easily determine whether patients have diseases such as cerebral infarction and tumors, and quantitatively analyze their location, size, and morphology, thus providing a more reliable basis for diagnosis.

In addition, image segmentation can also be used to extract feature parameters of specific regions, such as grayscale values, shape features, etc., to assist physicians in quantitative analysis and disease assessment. Therefore, image segmentation has a significant impact on the effectiveness of imaging diagnosis, providing more accurate and comprehensive image information, providing physicians with a more diagnostic basis, and helping to improve the early diagnosis and treatment of diseases.

(5) Technical factors

Imaging quality may be affected by technical factors such as patient movement, metal artifacts (in the case of MRI), and imaging parameters. Patient movement can cause blurring or artifacts in the image, reducing image quality and diagnostic accuracy. Especially in imaging processes such as head MRI that require a long scanning time, the patient’s discomfort or movement can lead to image blurring or artifacts, affecting the doctor’s accurate identification of lesions. Metal artifacts are image abnormalities caused by metal substances in the patient’s body that may obscure important anatomical structures or areas of the lesion. In MRI imaging, metal artifacts may reduce the clarity of brain structures and interfere with the doctor’s diagnosis [17]. Additionally, the selection of imaging parameters will directly affect the image quality. For example, the setting of scanning time affects the clarity of the image and the motion tolerance. The choice of resolution and contrast directly affects the display of image details and the recognition of lesions. Therefore, medical personnel must pay attention to optimizing imaging parameters and controlling the impact of technical factors on imaging quality when performing imaging diagnosis, to achieve the best diagnostic effect.

(6) Patient Factors

Patients may have certain diseases or implants, which may make certain imaging methods unsuitable or have contraindications. For example, for patients with pacemakers or artificial heart valves, there may be contraindications to MRI imaging, as strong magnetic field and radio frequency pulses in MRI may have adverse effects on the implant [18]. For patients with metal implants, such as artificial joints or metal tooth fillers, metal artifacts may occur, affecting image quality and diagnostic effectiveness. Additionally, the patient’s body size and position may also affect the image quality, such as in the multi-slice CT scan of obese patients, as excess adipose tissue can interfere with the clarity of the image.

(7) Title or atypical presentations

Subtle or atypical symptoms can have a significant impact on the effectiveness of imaging diagnosis. Some strokes may show atypical symptoms, making it difficult to accurately diagnose them by imaging. For example, some small or localized cerebral infarctions may manifest as mild sensory abnormalities, language disorders, or cognitive decline, without typical symptoms such as facial paralysis or paralysis of the limb. In this case, even if imaging examinations such as head CT or brain MRI are performed, the lesions may not be obvious enough or localized, resulting in limited imaging diagnostic effectiveness [19]. In addition, some other neurological or metabolic diseases can also exhibit symptoms similar to stroke, such as epilepsy, migraines, hypoglycemia, etc., further increasing the complexity of diagnosis. Therefore, for patients with subtle or atypical symptoms, a comprehensive clinical evaluation is necessary.

### High-frame rate imaging algorithms

In the prediction of acute cerebral infarction using Cmmi-MHR combined with thromboelastography parameters, high frame rate imaging algorithms can provide more detailed time series images, capture the dynamic process of thrombus formation and dissolution, and help to accurately assess the speed and range of development of the infarction. By combining Cmmi-MHR with thromboelastography parameters, we can comprehensively evaluate the morphology and histological characteristics of thrombi based on their elastic characteristics, providing more accurate information for predicting the size of the infarct and the prognosis of the patient.

By using high frame rate imaging algorithms to achieve faster image acquisition speed and higher temporal resolution, more precise blood flow dynamic information is provided to evaluate the stability of collateral circulation and the elastic characteristics of thrombus tissue, providing a more comprehensive assessment of the risk of cerebral infarction. This comprehensive application combines imaging technology with the biological characteristics of diseases, which can improve the accuracy of early diagnosis and prediction of acute cerebral infarction and provide stronger support for personalized treatment.

#### Beamforming algorithm - Direct Attached Storage (DAS) spatial compounding algorithm for open systems

**Fig. 1:**
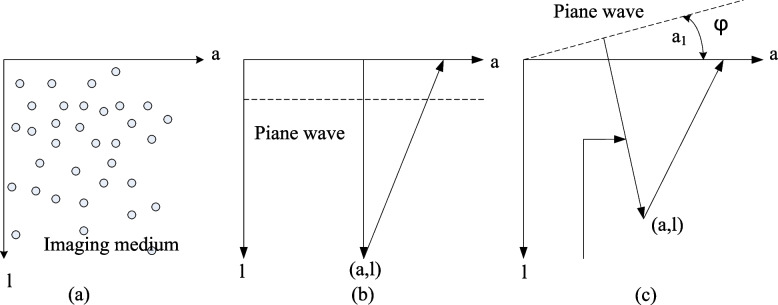
Schematic diagram of the array and AL plane image

Generally, a schematic diagram of the construction of an ultrasound imaging system is shown in Fig. 1 [20]. A multi-array element (typically 128 channels) is placed on the imaging medium through the coupling agent, the A direction is parallel to the array, and the L direction is parallel to the beam transmission direction, representing the imaging depth. In plane-wave imaging, all elements of the detector array are first excited to form a set of wave arrays that are approximately plane in the medium. Each array element would receive the composite echo from each scattering point of the medium [21, 22]. Because in plane wave mode, this paper does not focus when transmitting, this paper superimposes the echo signals of each unit on each scattering point to obtain $KP(a_{1} ,d)$.

In the unbiased plane-wave emission mode, that is, the plane wave with an angle of 0, its wavefront is parallel to the a-axis. In the two-way echo time process, the ultrasonic waves are sent to the scattering point $(a,l)$, and then received by the scattering point. The echo path is as follows:

1 $$\beta (a_{1} ,a,l) = (l + \sqrt {{{l^{2} + (a - a_{1} )^{2} } \mathord{\left/ {\vphantom {{l^{2} + (a - a_{1} )^{2} } e}} \right. \kern-0pt} e}}$$

Here e is the velocity constant defined in this article in the propagation medium [23].

Each imaging aperture unit obtains the backscattered signals of the scatterers $(a,l)$ of the pixels in the target imaging area and superimposes these backscattered signals on the array direction 1 [24], as shown in Fig. 1. The signal value of the pixel point represented by the right formula can be obtained:

2 $$w(a,l) = \int_{a - x}^{a + x} {KP(a_{1} ,\beta (a_{1} ,a,l))ta_{1} }$$

In Fig. 1a, in the AL direction, a represents the depth direction, and l represents the direction of the array. In Fig. 1b, without angular offset, the transmission channel for point $(a,l)$ in the medium is 1, and the reception channel is $\sqrt {l^{2} + (a - a_{1} )^{2} }$. In Fig. 1c When the transmit deflection angle is $\phi$, the transmit path of the calculated $(a,l)$ point is: $l\cos \phi + a\sin \phi$ and the receive path is $\sqrt {l^{2} + (a - a_{1} )^{2} }$.

**Fig. 2:**
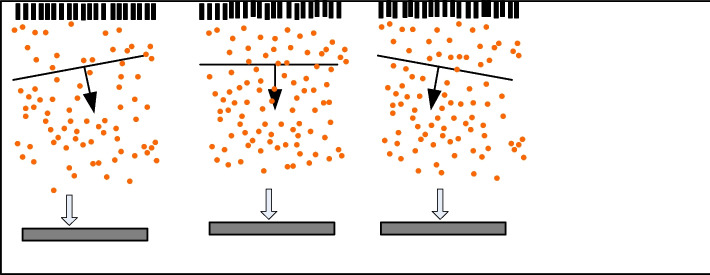
Schematic diagram of three-angle continuous plane wave image composite

Aperture 2 × represents the number of array elements used for one image. A modulated KP scan line signal can be obtained by superimposing different delay functions $\beta (a_{1} ,a,l)$ on the pixel points at each aperture position. When the 0-angle plane wave is emitted, this article does not focus on the image object, resulting in image contrast and signal-to-noise ratio [25, 26]. To improve image quality, a synthetic imaging method is proposed, as shown in Fig. 2. This method uses successive plane wave images emitted from different angles [27]. Figure 2 shows the basic principle of spatial composite imaging.

The time for the wavefront from emission to contact with a point $(a,l)$ in the imaging medium is:

3 $$\beta_{ce} (\phi ,a,l) = \frac{l\cos \phi + a\sin \phi }{e}$$

Here, $\phi$ is the angle between the plane wave and the surface of the array element [28, 29].

The time for the reflected echo to reach the $a_{1}$ position is:

4 $$\beta_{kce} (a_{1} ,a,l) = \frac{{\sqrt {l^{2} + (a - a_{1} )^{2} } }}{e}$$

The two-way launch schedule is:

5 $$\beta (\phi ,a_{1} ,a,l) = \beta_{ce} + \beta_{kce}$$

Then, similar to the radiation of the 0-angle plane wave, this paper iterates the backscattered signal of each scatterer, thus achieving the delayed iteration [30].

#### Beamforming algorithm - frequency beam migration (f-k migration) algorithm

**Fig. 3:**
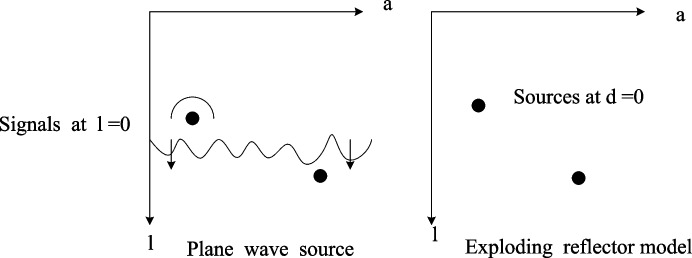
Schematic diagram of the explosion reflection model

It is generally believed that the propagation of the plane wave in the medium is that the wavefront meets the scattering particles of the medium and then forms a spherical echo, which is then received by the ultrasonic array element. However, in the DAS method, the scatterers in the medium are calculated one by one to obtain the beam-formed signal, which requires a lot of computing resources. This method is first used for seismic detection, and the premise of using it for plane wave ultrasonic beamforming is based on an explosive reflection model (ERM). The ERM model ignores the propagation of the plane wave and assumes that it is a hyperbolic signal caused by the spontaneous vibration of the scatterer, as shown in Fig. 3. The advantage is that in the frequency domain, the signal operation at the hyperbolic attenuation point can be effectively reduced, thereby improving the operation speed.

Therefore, compared to the DAS method, it is necessary to match the two-time histories of ultrasound in the medium with the time course of the explosion mode. First, this article will start with the unbiased launch of plane waves. Then the scatter point received by the array element at position a on the sensor:

6 $$\beta_{w} (a) = (l_{w} + \sqrt {{{l_{w}^{2} + (a_{w} - a)^{2} } \mathord{\left/ {\vphantom {{l_{w}^{2} + (a_{w} - a)^{2} } e}} \right. \kern-0pt} e}}$$

Here e is the propagation velocity of the sound wave in the medium.

According to the principle of the explosion model, the echo length of a line from the explosion of the scattering point to the signal received by the array element is as follows:

7 $$\hat{\beta }_{w} (a) = \sqrt {{{\hat{l}_{w}^{2} + (\hat{a}_{w} - a)^{2} } \mathord{\left/ {\vphantom {{\hat{l}_{w}^{2} + (\hat{a}_{w} - a)^{2} } {\hat{e}}}} \right. \kern-0pt} {\hat{e}}}}$$

In blast mode, $\hat{e}$ is its speed of sound. The signal received by the array element at the $a = a_{w}$ position in the array element is the strongest. At this time, let $\hat{\beta }_{w} (a) = \beta_{w} (a)$, that is, people can get:

8 $$\hat{e} = \frac{\sqrt 2 }{2}e,\hat{l}_{w} = \sqrt 2 l_{w}$$

By changing the coordinates, the wave path of the explosion mode can be compared with the time of the two wave paths.

**Fig. 4:**
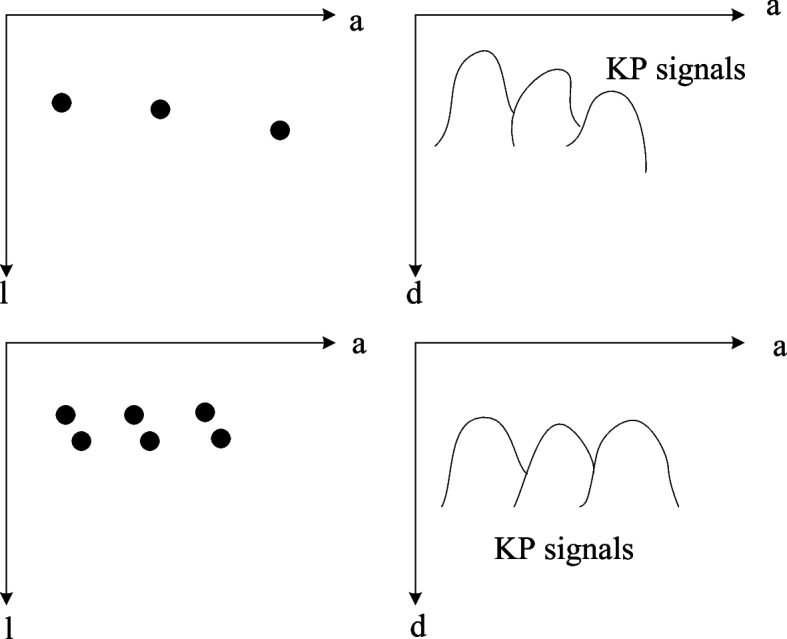
Schematic diagram of explosion model calculation when angled plane waves are launched

Next, this article will discuss how to calculate the wave path of the explosion mode with angular offset. As can be seen in Fig. 4, when the deflection angle of the plane wave is $\alpha$, the actual wave path time is

9 $$\beta_{w} (a) = \frac{1}{e}(\sin (\alpha )a_{w} + \cos (\alpha )l_{w} + \sqrt {l_{w}^{2} + (a_{w} - a)^{2} } )$$

The basic assumption of the f-migration method is that the echo of the scattering point encountered by the sound wave in the medium is a hyperbolic wave. Among them, the peak signal of the hyperbolic wave is the largest, and the others are attenuated, so only the peak of the hyperbolic wave is used at this point. Therefore, in this paper, the attenuation signal caused by the angular offset should be removed first, and the position of the down-scattering point would move down as a whole, and the wave path time would become as shown in the following formula:

10 $$\beta_{w} (a) = \frac{1}{e}(\sin (\alpha )(a_{w} - a) + \cos (\alpha )l_{w} + \sqrt {l_{w}^{2} + (a_{w} - a)^{2} } )$$

Set a set of constant parameters $(\phi ,\eta ,\chi )$, let:

11 $$\left\{ \begin{array}{lll} \hat{e} = xe \hfill \\ \hat{l}_{w} = \eta l_{w} \hfill \\ \hat{a}_{w} = a_{w} + \chi l_{w} \hfill \\ \end{array} \right.$$

The explosion model time history becomes:

12 $$\hat{\beta }_{w} (a) = \frac{1}{\phi e}\sqrt {(a_{w} + \chi l_{w} - a)^{2} + \eta^{2} l_{w}^{2} }$$

Matching formulas (10) and (12), the same text makes $(a = a_{w} = \hat{a}_{w} )$ and calculates the constant parameters as:

13 $$\left\{ \begin{array}{lll} \phi = \frac{1}{{\sqrt {(1 + \cos (\alpha ) + \sin^{2} (\alpha ))} }} \hfill \\ \eta = \frac{{(1 + \cos (\alpha ))^{{{\raise0.7ex\hbox{$3$} \!\mathord{\left/ {\vphantom {3 2}}\right.\kern-0pt} \!\lower0.7ex\hbox{$2$}}}} }}{{1 + \cos (\alpha ) + \sin^{2} (\alpha )}} \hfill \\ \chi = \frac{\sin (\alpha )}{{2 - \cos (\alpha )}} \hfill \\ \end{array} \right.$$

This paper finds that at 0, that is, there is no deflection angle at launch, $(\phi, \,\eta, \,\chi ) = (\frac{\sqrt 2 }{2},\sqrt 2 ,0)$

One of the most critical points is to use the Fourier transform to find the exact solution of the wave formula, which is the simplest and fastest method known at present. This paper enumerates several major steps in the transformation of KP signals from the time domain to the frequency domain R space.

14 $$\lambda (a,l,d) = \int {\int_{ - \infty }^{ + \infty } {\gamma (r_{a} ,l,g)c^{{2j\pi (r_{a} a - gd)}} tr_{a} tg} }$$

Among them, $\lambda (a,l,d)$ represents the two-dimensional blast mode wavefield in the time domain, and $\gamma (r_{a} ,l,g)$ represents the Fourier transform from the time domain to the frequency domain $\lambda (a,l,d)$. $r_{a}$ and g represents the current wavenumber and current frequency at position a in the r space, respectively. In r space, the wave formula satisfies the Helmholtz formula:

15 $$\frac{{\varepsilon^{2} \gamma }}{{\varepsilon l^{2} }} + 4\pi^{2} \hat{r}_{l}^{2} \gamma = 0$$

Substituting the converted wave speed into this paper, the converted spectral wave number is obtained:

16 $$\hat{r}_{l}^{2} = \frac{{g^{2} }}{{\hat{e}^{2} }} - r_{a}^{2}$$

The boundary condition of formula (15) is $\gamma (r_{a} ,0,g)$, that is, the frequency region: $\lambda (a,l = 0,d)$ that is to say, after determining the boundary on the wave field, after the explosion model moves, it can be expressed as:

17 $$\lambda (a,l,0) = \int {\int_{ - \infty }^{ + \infty } {\gamma (r_{a} ,0,g)c^{{2j\pi (r_{a} a - \tilde{r}_{l} l)}} tr_{a} tg} }$$

A variable $\hat{r}_{l}$ is introduced, and the conversion form of this variable is as follows.

18 $$g(\hat{r}_{l} ) = \hat{e}sign(\hat{r}_{l} )\sqrt {l_{a}^{2} + \hat{r}_{l}^{2} }$$

The migrated wavefield can be represented by the frequency domain function $(r_{a} ,\hat{r}_{l} )$:

19 $$\lambda (a,l,0) = \int {\int_{ - \infty }^{ + \infty } {\frac{{\hat{e}\hat{r}_{l} }}{{\sqrt {r_{a}^{2} + \hat{r}^{2} } }}\gamma (r_{a} ,0,g(\hat{r}_{l} ))c^{{2j\pi (r_{a} a - \tilde{r}_{l} l)}} tr_{a} t\hat{r}_{l} } }$$

Therefore, the final solution of f-migration is obtained according to the Fourier transform.

## Predictive value of MHR, monocytes, and high-density lipoprotein on clinical outcomes of patients with acute cerebral infarction

### Experimental design

#### Research objects

This article is a hospital-based retrospective study of patients with acute ischemic stroke who were admitted to the Neurosurgery Department of Hospital A between April 2021 and April 2022 based on a unified clinical registry.

Inclusion criteria: over 18 years of age; and according to the diagnostic criteria determined by the 2021 “China AIDS diagnosis and Treatment Standards”; 24 h or more, signs of local neurological loss; Head CT or MRI was performed within 72 h of hospitalization, and the corresponding cerebral infarction was found.

Exclusion criteria: Suffering from other neurological diseases that significantly affect the research results, such as brain tumors, brain injuries, etc.; Patients with severe cardiovascular disease, liver and kidney dysfunction, or other important organ dysfunction.

Subject conditions: hospitalization 72 h after onset; fatal brain stem infarction; incomplete clinical or laboratory data.

Of the 951 eligible patients, 14 patients with a lack of laboratory data, 116 patients with 72 h of onset, 5 patients with severe and fatal brain stem infarction and 13 patients with missing follow-up data were excluded, and 803 patients were eligible for inclusion.

#### Collecting clinical data

All patients used a standard clinical registration form to collect relevant data such as age, sex, medical history, and clinical characteristics at the time of admission, during hospitalization, and after discharge.

The diagnosis is based mainly on the following: the main basis for the diagnosis of hypertension: multiple measurements of the patient’s right upper extremity or the healthy side, diastolic blood pressure greater than 90 mmHg, the use of antihypertensive drugs or a history of hypertension;

The condition of a smoking history is smoking or regular smoking (20 cigarettes per day for more than 1 year).

History of drinking: more than 50 g per day or 500 g per week.

There was a history of cerebral infarction, cerebral hemorrhage, etc., without any sequelae.

Clinical characteristics: NIHSS was used to evaluate neurological function scores at the time of hospitalization. The modified Rankin Scale (mRS) was used to evaluate the prognosis of patients after discharge.

In the laboratory test, 2 ml of venous blood was collected by an EDTA-k2 anticoagulation vacuum tube, mixed with an automatic blood cell analyzer XE-5000 production line to measure the number of leukocytes and monocytes, and 5 ml of venous blood was taken on an empty stomach the next morning after hospitalization. All patients were tested 24 h after hospitalization.

According to the grading criteria of the Online Certificate Status Protocol (OCSP), patients were included in head CT or magnetic resonance imaging within 72 h and divided into 4 categories according to clinical manifestations: total anterior circulation infarction (TACI); partial anterior circulation infarction (PACI); posterior circulation infarction (POCI); lacunar infarction (LACI).

#### Results

1. Stroke severity score

The severity of the stroke was determined mainly by the NIHSS score at the time of hospitalization, and the NIHSS score of 0–3 belonged to mild cerebral infarction.

2. Diagnostic criteria for pulmonary infections

Post-stroke pulmonary infection is based on the diagnostic criteria for stroke-related pneumonia in 2021.

The details are as follows: At least one condition is met: body temperature > 38 °C for no other reason; decreased white blood cell count (< 4 × 109/L) or increased white blood cell count (> 11 × 109/L).

3. Inspection method

Combined X-ray or lung CT: New or evolving exudate, consolidation, or hole.

4. Evaluation of prognostic effects

Poor prognosis at discharge: When a patient is discharged, if mRS < 2, the patient has a good prognosis, while mRS > 2 indicates a poor prognosis.

5. Confirmation of death cases

For 90 days, phone or face-to-face tracking of all deaths, deaths need to wait for confirmation from family members, colleagues, or death certificates, medical records, etc.

### Results

#### Basic characteristics of the included patients

The underlying characteristics of the patients were analyzed based on the MHR quartiles at admission (Q1: < 0.21; Q2: 0.21–0.29; Q3: 0.3–0.45; Q4: > 0.45) (Table 1). Compared to the MHR group of the first quartile, patients with MHR in the fourth quartile had higher proportions of men, a history of smoking, and a history of hypertension. WBC counts, neutrophil counts and monocyte counts were higher in quartile 4 MHR patients. Other baseline data such as age, heart rate, baseline NIHSS score (Table 2), history of diabetes, history of coronary heart disease, history of cerebral infarction, rate of intravenous thrombolysis (Table 3), classification of OCSP (Table 4) were not significant differences.

**Table 1:** Basic characteristics of the patient by MHR quartile analysis at admission (a)

/	Q1	Q2	Q3	Q4	/
Characteristics	< 0.21	0.21–0.29	0.30–0.45	> 0.45	*P*-value
Number of subjects	196	204	200	203	/
Age	70.1 ± 12.6	69.0 ± 11.6	69.6 ± 12.3	68.3 ± 12.6	0.507
Current smoking (%)	41 (20.9)	52 (25.5)	57 (28.5)	68 (33.5)	0.038
Gender (%)	79 (40.3)	91 (44.6)	116 (58)	145 (71.4)	< 0.001
Current drinking (%)	31 (15.8)	47 (23)	47 (23)	46 (22.7)	0.196

**Table 2:** Basic patient characteristics by MHR quartile analysis at admission (b)

Heart rate (beats/min)	74.6 ± 10.3	75.3 ± 10.5	74.8 ± 10.9	76.1 ± 12.6	0.504
WBC (10^9^/L)	5.9 ± 2.2	6.3 ± 1.9	7.1 ± 2.2	8.8 ± 6.9	< 0.001
Monocytes (10^9^/L)	0.26 ± 0.09	0.35 ± 0.07	0.44 ± 0.10	0.61 ± 0.20	< 0.001
TC, mmol/L	4.7	4.7	4.5	4.2	< 0.001
LDL-C, mmol/L	2.7	2.7	2.7	2.5	0.008
HDL-C, mmol/L	1.6	1.4	1.2	1.0	< 0.001
FPG, mmol/L	5.6	5.4	5.6	5.5	0.826
Baseline NHSS score	4	4	4	5	0.556

**Table 3:** Basic patient characteristics by MHR quartile analysis at admission (c)

Hypertension	120 (61.2)	139 (68.1)	157 (78.5)	149 (73.4)	0.001
Diabetes	41 (20.9)	45 (22.1)	54 (27)	50 (24.6)	0.485
Coronary heart disease	12 (6.1)	7 (3.4)	8 (4.0)	15 (7.4)	0.244
Atrial fibers	28 (14.3)	29 (14.2)	23 (11.5)	36 (17.7)	0.363
History of stroke	48 (24.5)	52 (25.5)	48 (24)	48 (24)	0.976
Intravenous thrombolysis	8 (4)	8 (4)	7 (3.5)	6 (3)	0.932

**Table 4:** Patient characteristics by quartile analysis of MHR at admission (d)

Quartile Analysis	TACI	PACI	POCI	LACI
Q1	13 (6.2)	78 (39.4)	36 (18.0)	73 (36.8)
Q2	8 (3.6)	89 (43.2)	25 (11.9)	86 (41.7)
Q3	82 (5.6)	82 (40.6)	38 (18.6)	72 (35.6)
Q4	11 (5.1)	81 (40.1)	38 (18.3)	76 (37.5)

#### Correlation of MHR, monocyte count, and HDL with the NIHSS score at the time of hospitalization

The study showed that monocyte count and HDL were significantly correlated with the NIHSS score during hospitalization (*r* = 0.131 *P* < 0.001); *r* = 0.094 *P* = 0.008). There was no significant correlation between MHR and NIHSS score at admission (*r* = 0.053 *P* = 0.132) (Table 5).

**Table 5:** Spearman correlation analysis of MHR, monocytes, and HDL and admission NIHSS score

/	NIHSS	/
/	*r*	*P* value
HDL	0.094	0.008
MHR	0.053	0.132
monocytes	0.131	< 0.001

**Fig. 5:**
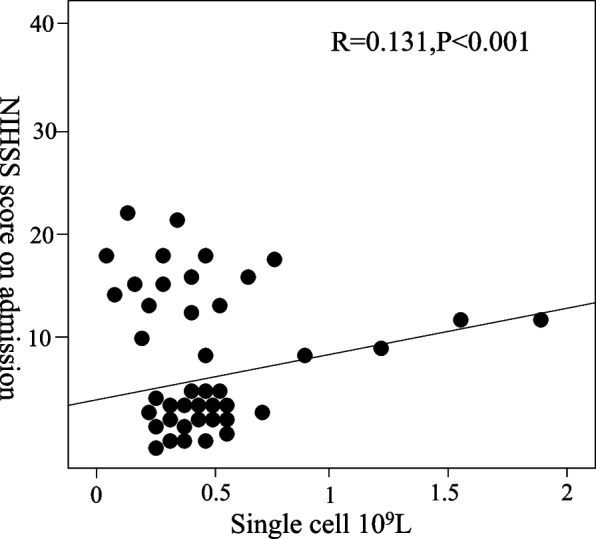
Scatter plot of the correlation analysis between monocytes and the NHISS score on admission

**Fig. 6:**
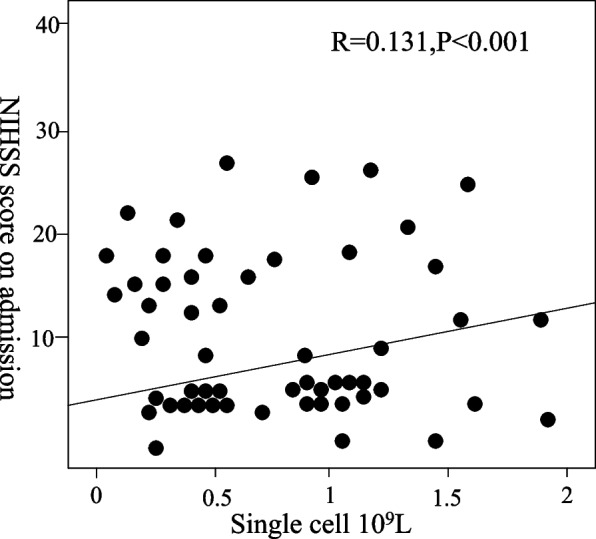
Scatter plot of the correlation analysis between HDL and the NHISS score at admission

**Fig. 7:**
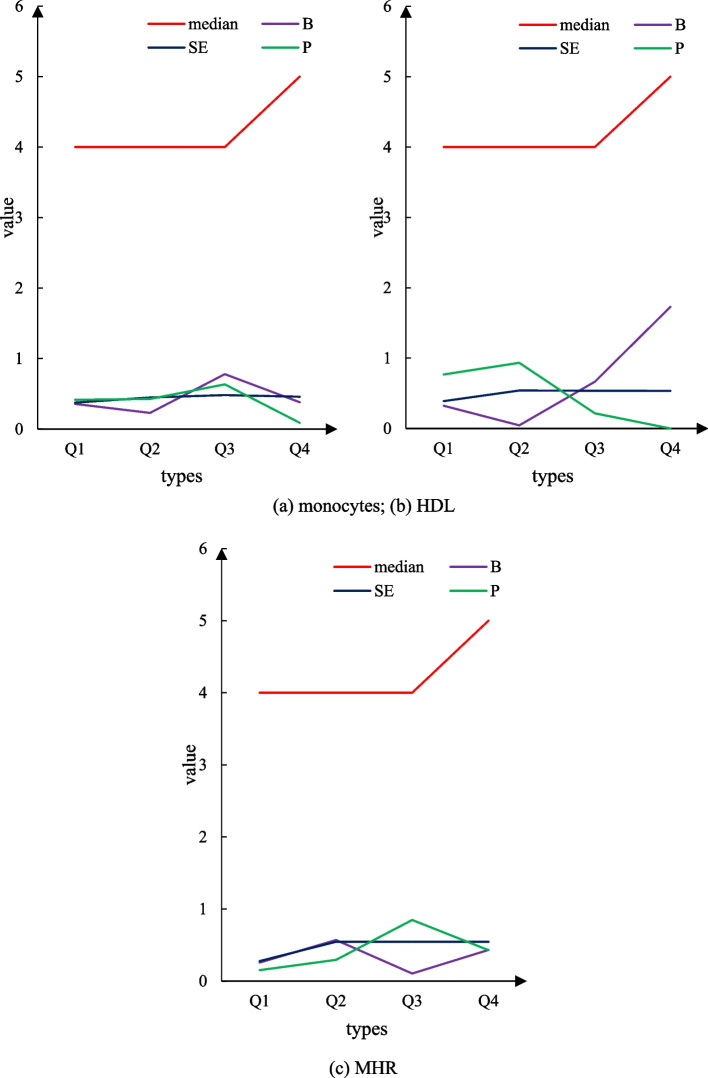
Correlation of MHR, monocyte count, and high-density lipoprotein with the NIHSS score on admission. **a** shows the correlation between monocytes and the NIHSS score. **b** shows the correlation between HDL and NIHSS scores. **c** shows the correlation between the MHR and the NIHSS scores

According to the comparative description of Figs. 5 and 6, other factors were analyzed by multiple linear regression, and the results showed that the NHISS score was significantly higher than that of the first quantile group at the time of hospitalization (*P* = 0.001). Figure 7a shows the correlation between single nuclei and the NIHSS score on admission, Fig. 7b shows the correlation between HDL and the NIHSS score on admission, and Fig. 7c shows the correlation between MHR and the NIHSS score on admission. However, the results did not show significant differences in the NHISS scores between the quantile groups of HDL, MHR, and monocytes (Fig. 7).

#### The relationship between MHR, monocyte count, HDL, and discharge outcome

**Fig. 8:**
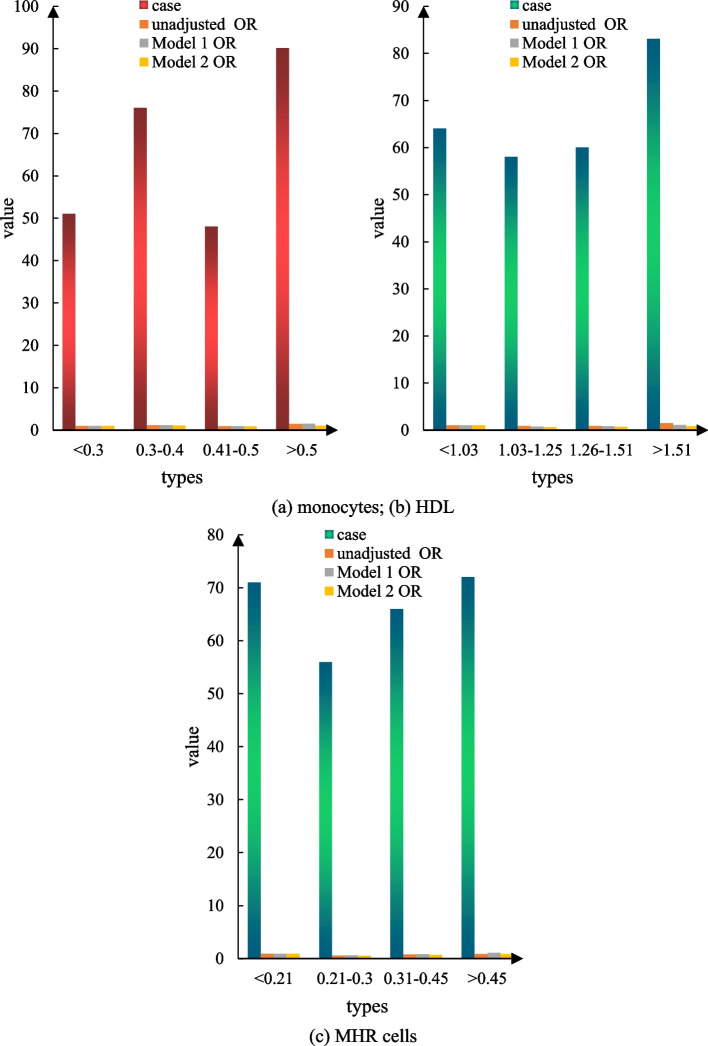
Association of MHR, monocyte count, and HDL with discharge outcomes. **a** shows the relationship between monocytes and discharge outcomes. **b** shows the relationship between HDL and discharge results. **c** shows the relationship between MHR cells and discharge outcomes

**Fig. 9:**
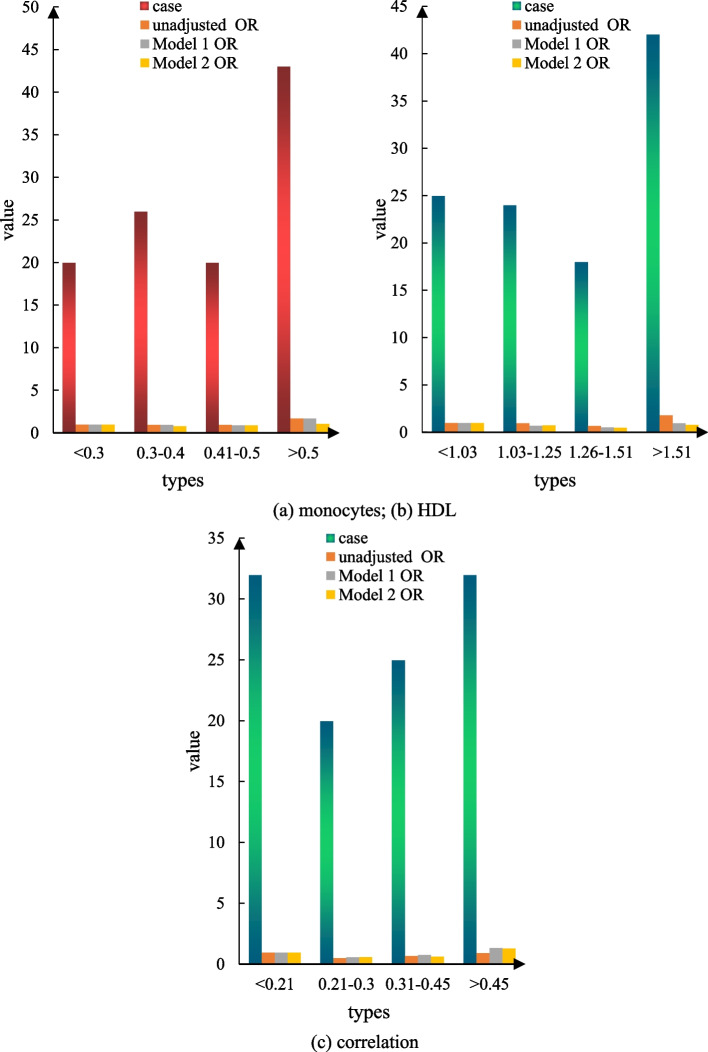
MHR, monocyte count, and high-density lipoprotein are associated with 90-day mortality. **a** shows the correlation analysis between monocytes and the 90-day mortality rate. **b** Correlation analysis between HDL and 90-day mortality rate. **c** Correlation analysis between MHR and the 90-day mortality rate

Figure 8a is the correlation between the nucleus of a single cell and the discharge outcome, Fig. 8b is the correlation between HDL and the discharge outcome, and Fig. 9c is the correlation between MHR and the discharge outcome. In this trial, 265 patients had mRs > 2 at discharge, and 33.0% of the patients had a poor prognosis at discharge. The results of the single-factor logistic regression analysis showed that there was no significant difference in the prognosis of MHR, monocyte count, and HDL during hospitalization compared to the 1-quantile group (Fig. 8).

#### Association of MHR, monocyte count, and HDL with death within 90 days

Figure 9a is the correlation between monocytes and 90-day death, Fig. 9b is the correlation between HDL and 90-day death, and Fig. 9c is the correlation between MHR and 90-day death. All included patients had all-cause death after 90 days, and 109 patients, or 13.6% of all patients, died during the follow-up period. The results of the univariate logistic regression analysis showed that HDL (OR = 1.8995, trend *P* = 0.049) had a probability of dying within 90 days (OR = 0.81, 95% CI = 1.06–3.11, trend *P* = 0.523) during hospitalization. There was no significant difference in mortality after 90 days. There were no significant differences in the risk of MHR or monocyte count within 90 days of hospitalization, regardless of whether or not adjustment for confounders such as age, sex, and NIHSS score was performed (Fig. 9).

To further validate the results of the statistical analysis of the relationship between MHR, monocyte count, high-density lipoprotein, and death within 90 days in this study, support vector machines were used to predict the mortality risk of patients with monocytes, HDL, and MHR. The final results of the risk index are shown in Table 6:

**Table 6:** Risk index results

Sequence	Classification	Risk index	*P* value
1	monocytes	0.213	0.514
2	HDL	0.204	
3	MHR	0.209	

From the results of the risk index and the *P* value, there was no significant difference in the risk of hospitalization within 90 days for HDL, MHR, or monocytes.

### Discussion

The results showed that MHR and monocyte counts at admission were not associated with neurological severity at admission, poor prognosis at discharge, and death within 90 days. There was a weak correlation between HDL and neurological severity at hospitalization, and MHR and monocyte count at hospitalization had a certain relationship with the appearance of poststroke-related pneumonia. Inflammation is a key factor in the occurrence and development of ischemic stroke [31]. Inflammation not only accelerates the occurrence of stroke but also worsens the condition of stroke. In the occurrence and development of most strokes, inflammation is also involved in most strokes.

Endothelial dysfunction is the first stage of atherosclerosis, and when endothelial function is abnormal, activated monocytes interact with damaged endothelial cells. Existing studies have shown that the enhanced activity of MMPA can accelerate the destruction of the vascular elastic membrane, thereby causing plaque rupture. Monocytes, a specific type of atherosclerosis, interact with vascular endothelial cells and platelets to cause vascular inflammation, endothelial dysfunction, and vascular occlusion, which can predict plaque development [32]. Our findings suggest that high monocyte counts are associated with plaque formation in ST-segment elevation myocardial infarction. At the same time, the number of monocytes also has a certain relationship with the severity, prognosis, and mortality of coronary heart disease, stroke, and other related diseases. Animal experiments showed that 12 h after intracerebral hemorrhage, monocytes invaded and surrounded the hematoma, reaching a maximum value in 5 days.

Several recent studies have confirmed that high monocyte counts are strongly associated with mortality at 30 days from intracerebral hemorrhage [33]. A study in the United States showed that high monocyte counts at the time of hospitalization were not associated with the number of hematomas in cerebral hemorrhage, but were not associated with 30-day mortality. Patients with high monocyte counts at admission had a significantly higher 30-day mortality rate. However, some studies have indicated that after acute cerebral infarction after 3 months, monocyte count is not associated with a poor prognosis.

In recent years, several experiments have shown that there is a close relationship between HDL and monocytes, and HDL can regulate the activation, adhesion, and migration of monocytes [34]. Studies have shown that HDL can inhibit the migration of monocytes to the inner layer. Subsequently, HDL and its main protein component, apolipoprotein A-1, can inhibit the activity of CD11b, thus inhibiting the inflammatory response of human monocytes. Furthermore, HDL may also increase indirect effects such as anti-inflammatory and antioxidant, thus enhancing antioxidant capacity. Several recent studies have shown a new view that HDL is anti-inflammatory. HDL plays an anti-inflammatory role in the hematopoietic system, it can promote the proliferation of hematopoietic stem cells, and it can inhibit the production of monocytes.

More clinical studies have shown that high HDL can reduce the area of ischemic stroke damage and reduce the severity of the disease, thus improving the prognosis of patients. Therefore, the high-density lipoprotein content and its associated monocyte count are of great significance in the occurrence, development, severity, and prognosis of stroke. MHR can be used as a new indicator to reflect the inflammatory response, and multiple studies have shown that there is a strong link between high MHR and the occurrence and death of cardiovascular events. 513 cases of acute ST-segment elevation myocardial infarction and percutaneous coronary intervention (PCI) were analyzed. The results showed that there is a certain correlation between high MHR and serious adverse cardiovascular events (MACEs) and death after PCI. A survey of patients preparing for coronary angiography showed that an increase in MHR was associated with a 2.03-fold increase in the incidence of MACE. Recently, a new study has shown that high MHR is strongly associated with the prognosis and mortality of cerebral infarction. The team in this article found that elevated MHR was strongly associated with patient discharge, prognosis, and death 3 months later [35]. In patients with ischemic cerebral infarction, high MHR is an independent predictor. However, we found only a weak association between HDL in the hospital and neurological severity in the hospital, and no associations between monocyte count and MHR with severity in the hospital, discharge outcomes, and death within 90 days.

The function of monocytes after cerebral ischemia is bidirectional and the mechanism is closely related to the type of monocyte. Because the experiment only counted monocytes, not monocytes, the relationship between monocyte severity at hospitalization, hospital discharge, and 90-day mortality was not available. In this article, monocytes will be classified and their functions analyzed.

Dynamic changes in monocytes after ischemic cerebral ischemia may be related to the time when monocytes and macrophages invade brain damage after cerebral ischemia. Several studies have shown that neutrophils in peripheral blood enter brain tissue in about 1 day, while monocytes and macrophages in peripheral blood are more abundant in 3 to 7 days. All patients included in this study were in 72 h and the monocytes in the peripheral blood probably did not change. According to the dynamic characteristics of monocytes, the relationship between the two can be obtained by appropriately prolonging the onset time of selected patients.

The results of this paper suggest the appearance of immunosuppressive syndrome after stroke. In the acute phase of cerebral infarction, due to the activity of sympathetic nerves, the number of immune cells decreases and also stimulates the increase in inflammatory cells such as neutrophils and monocytes, resulting in the secretion of glucocorticoids and the onset of SAP. At the same time, after acute cerebral infarction, parasympathetic and pituitary-adrenal axis abnormalities also occur. Activation of parasympathetic nerves, in particular, induces cholinergic activity, which leads to the production of systemic applications and products (SAP). Therefore, by detecting the monocyte count and MHR of patients during hospitalization, the risk of stroke-related pneumonia can be better judged, and the basis for the early diagnosis and treatment of post-stroke pneumonia can be provided.

This article is primarily a single-center retrospective study and, although some study-relevant factors were identified in the multivariate analysis, other unmeasured or inappropriate factors could not be completely ruled out.

This article focuses on the Chinese population, but caution should be exercised for populations with other genetic backgrounds. At the same time, some patients with monocyte and HDL deficiency were excluded from the included cases, which would also cause inconsistent results.

### Research limitations and future research directions

This paper has a small sample size, but there are sample selection biases and limitations. Furthermore, there is a lack of long-term monitoring and observation data to evaluate the long-term impact of Cmmi-MHR combined with thromboelastography parameters on the prognosis of acute cerebral infarction. There are still certain technical limitations and standardization deficiencies in thromboelastography technology and Cmmi MHR calculation methods.

In future research, we will consider increasing the sample size, conducting long-term tracking studies, and delving into the stability and persistence of these parameters in prognosis prediction. We will also strive for technological improvements to improve image resolution and data accuracy, to further reveal the potential value of Cmmi-MHR combined with thromboelastography parameters in predicting acute cerebral infarction.

## Conclusions

Our study shows that there is a weak association between in-hospital HDL and in-hospital NIHSS. Although the association of HDL with clinical outcomes suggested that lowering HDL was associated with increased clinical outcomes, no statistically corresponding anti-inflammatory effect of HDL was found, thus reducing the pro-inflammatory effect of monocytes. The relationship between NLR, neutrophils, lymphocytes, and SAP has been confirmed, and the increase of NLR, neutrophils, lymphocytes, and other indicators can be used as early warning indicators of SAP. Fortunately, this article once again found the relationship between MHR, HDL, monocyte count, and SAP, which is helpful for the early assessment of SAP-related risk factors. The results showed that the increase in monocyte count was closely related to the appearance of SAP. The increase in monocyte count was associated with an increase in the incidence of SAP. After adjusting for other risk factors, when the number of monocytes was greater than 0.5 × 10^9^/L, the risk of SAP was 2.6 times lower than that of monocytes that did not reach 0.3 × 10^9^/L (*P* = 0.005). This suggests that monocyte count is an independent predictor of SAP. At the same time, the ROC curve was used to determine the role of monocyte count and MHR in stroke-related pneumonia, and it was found that the predictive effect of monocyte count was better than that of MHR.

## Data Availability

Data sharing did not apply to this article as no datasets were generated or analyzed during the current study. Data for this article were obtained from a clinical registry of patients with acute ischaemic stroke admitted to the Department of Neurosurgery at Hospital A from April 2021 to April 2022. The datasets generated and/or analyzed during the current study are not publicly available due to the data being not public but are available from the corresponding author upon reasonable request.
